# Off-pump total arterial bypass grafting for the elderly does not improve life expectancy

**DOI:** 10.3389/fcvm.2025.1598770

**Published:** 2025-05-01

**Authors:** Nuttapon Arayawudhikul, Ryohei Ushioda, Hideki Isa, Dit Yoongtong, Boonsap Sakboon, Jaroen Cheewinmethasiri, Thanin Lokeskrawee, Jayanton Patumanond, Suppachai Lawanaskol, Hiroyuki Kamiya

**Affiliations:** ^1^ Cardiovascular and Thoracic Surgery Unit, Department of Surgery, Lampang Hospital, Lampang, Thailand; ^2^ Department of Cardiac Surgery, Asahikawa Medical University, Asahikawa, Japan; ^3^ Department of Emergency Medicine, Lampang Hospital, Lampang, Thailand; ^4^ Center for Clinical Epidemiology and Clinical Statistics, Faculty of Medicine, Chiang Mai University, Chiang Mai, Thailand

**Keywords:** off-pump coronary artery bypass grafting, total arterial bypass grafting, multivessel coronary artery revascularization, elderly, coronary artery disease

## Abstract

**Objectives:**

To assess whether total arterial revascularization (TAR) offers survival and freedom from major adverse cardiac or cerebrovascular events (MACCE) benefit in elderly patients with multivessel coronary artery disease undergoing off-pump coronary artery bypass grafting (OPCAB), as compared to using a single internal thoracic artery (ITA) with additional saphenous vein graft (SVG).

**Methods:**

We retrospectively analyzed 598 patients aged >70 years who underwent coronary revascularization from August 2017–July 2023. After excluding high-risk patients and those with SVG plus more than two arterial grafts, 428 patients remained (101, TAR group; 327, single ITA + SVG group). A propensity score was used to create the TAR and single ITA + SVG groups with 1:1 ratio (100 patients in each group). Moreover, matching was performed based on eight covariates with preoperative clinical characteristics.

**Results:**

The unmatched cohort had 70 (69.3%) and 178 (54.4%) men in the TAR and ITA + SVG groups, respectively (mean age, 74.1 ± 3.5 and 75.2 ± 4.2 years, respectively). After matching, both groups had similar demographics. The survival (*p* = 0.410) and MACCE-free rates (*p* = 0.494) over 5 years were not significantly different between the two groups. Univariable analysis showed that TAR [hazard ratio (HR) = 0.74, 95% confidence interval (CI) = 0.44–1.22, *p* = 0.233] and complete revascularization (HR = 0.61, 95%CI = 0.34–1.09, *p* = 0.094) were not significant risk factors for long-term mortality.

**Conclusion:**

Elderly patients who underwent OPCAB with total arterial grafting did not show survival or free-MACCE benefits for over 5 years.

## 1 Introduction

To improve the long-term cardiac outcomes in isolated coronary artery bypass grafting (CABG), the use of arterial grafts, such as the radial artery (RA) or bilateral internal mammary artery (IMA), for the second most important non-left anterior descending (LAD) vessel has been recommended over the use of saphenous vein grafts (SVGs) (1). Recently, there has been debate about whether the extensive use of multiple arterial conduits (more than 2) offers additional mortality benefits as compared to CABG utilizing only two arterial grafts (2–6). Although observational studies and two meta-analyses have supported the use of three or more arterial grafts, including total arterial revascularization (TAR) (7, 8), the extent of this benefit across different age groups remains unclear. As a center in Southeast Asian countries that regularly performs TAR as off-pump coronary artery bypass grafting (OPCAB), our institution would like to explore its outcomes. Therefore, the present study aimed to assess whether TAR offers survival and freedom from major adverse cardiac or cerebrovascular event (MACCE) benefits in elderly patients with multivessel coronary artery disease who underwent TAR (OPCAB), as compared to grafting of the standard left internal mammary artery (LIMA) to the LAD with additional grafting to the other vessels using SVG in elderly patients.

## 2 Patients and methods

This single-center retrospective study enrolled all elderly patients who underwent isolated OPCAB due to a multivessel coronary artery disease in our institution from August 2017–July 2023. The study was conducted according to the guidelines stipulated in the Declaration of Helsinki and STROBE criteria of retrospective studies. The requirement for obtaining informed consent from the patients was waived owing to the retrospective nature of the study.

Patients were excluded from the study if they had undergone only SVG or SVG with more than two arterial grafts or had poor preoperative conditions (emergent and salvage cases or those with Society of Thoracic Surgeons score of >20).

**Figure 1:**
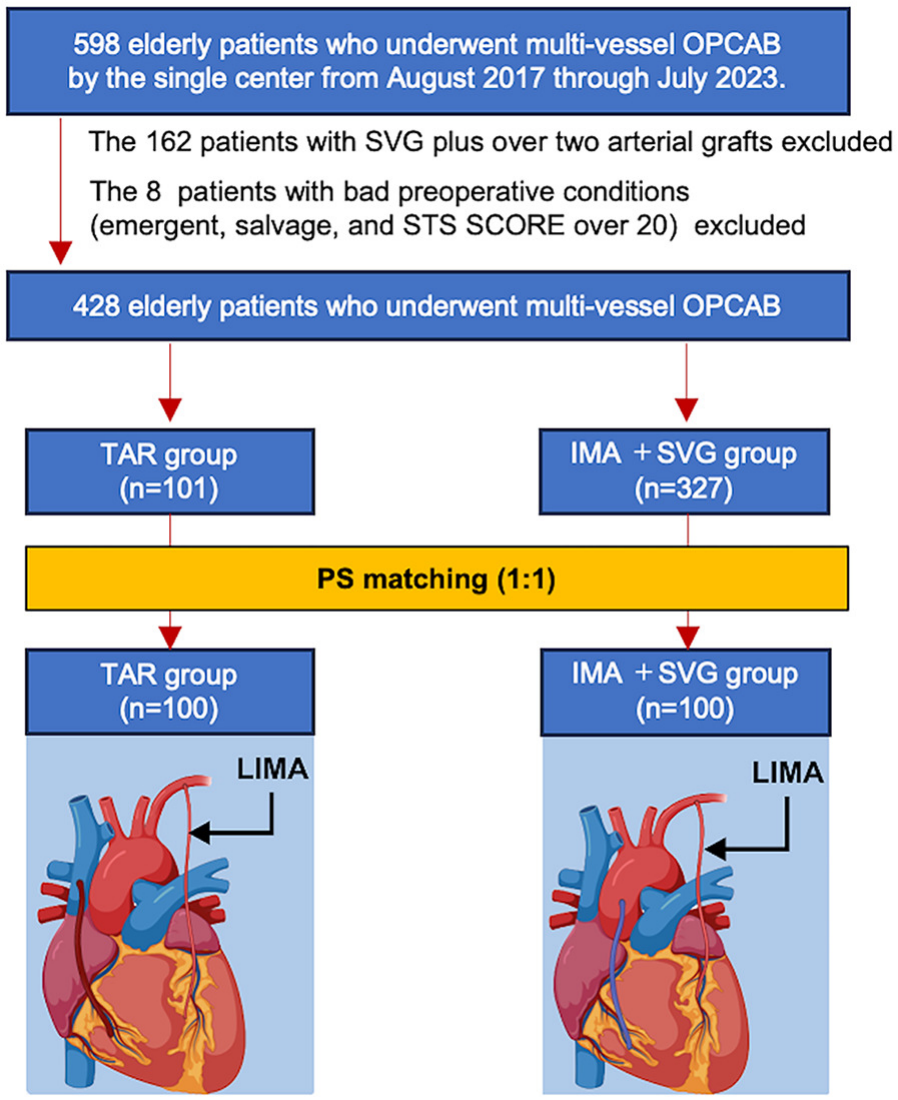
The protocol for this study.

The patients were divided into two unmatched groups (unmatched TAR and unmatched LIMA with SVG groups). Then, the TAR group was propensity score-matched (PSM) with the LIMA + SVG at a 1:1 ratio (TAR, *n* = 100; LIMA + SVG, *n* = 100), and matching was performed based on eight covariates with preoperative clinical characteristics (Figure 1). The choice between TAR and LIMA with SVG was made at the discretion of the attending surgeon. While there were no strict predefined criteria, TAR was generally selected for patients with relatively favorable preoperative conditions and suitable conduit anatomy. Follow-up data of all participants were collected in the planned outpatient clinic. The follow-up rate was 100%, and the mean follow-up duration was 1,491.6 ± 921.4 days.

### 2.1 Surgical procedures

OPCAB was performed regularly by four surgeons at our unit. An intra-aortic balloon pump was potentially used preoperatively in patients with critical left main and poor left ventricular ejection fraction. IMA and right gastroepiploic artery (RGEA) were harvested in a skeletonized fashion using an ultrasonic scalpel (Harmonic Scalpel, Ethicon Endosurgery, Cincinnati, OH), RA was harvested using electrocautery in a semi-skeletonized fashion, and SVG was also harvested using electrocautery non-touch technique. Heparin at a dose of 1 mg/kg was given before the division of the conduits and continued to maintain an activated coagulation time of >280 s through the entire operation. For the OPCAB procedure, a stabilizer (Octopus® tissue stabilizer, Medtronic, Minneapolis, MN, USA) was used to control the coronary targets. Three deep pericardial sutures were placed in the posterior pericardium at the left inferior pulmonary vein, medial to the inferior vena cava, and in between, exposing the lateral and inferior walls of the heart. To achieve a bloodless field, an intracoronary shunt and carbon dioxide blower were used. Transit-time flow measurement was used routinely after completing the anastomosis to validate graft patency.

### 2.2 Surgical technique for TAR

The most common configuration for TAR was *in situ* right internal mammary artery (RIMA) to the LAD, *in situ* LIMA to the obtuse marginal artery, and *in situ* RGEA to the posterior descending artery. The second approach was *in situ* LIMA to the LAD and Y-composite using free RA anastomosed to the LIMA for the rest of the target arteries. The last approach was *in situ* RIMAI composited using RA to the lateral and inferior areas.

### 2.3 Surgical technique for LIMA and SVGs

The first choice of SVG inflow was the ascending aorta. To ensure that the aorta is suitable for use, we consistently assessed its quality through a combination of preoperative and intraoperative evaluations, including chest x-rays showing the pulmonary artery in the upright and lateral views, preoperative noncontrast computed tomography, and intraoperative epi-aortic ultrasound scanning. The second choice was the IMA to achieve a no-touch technique. The choice of proximal anastomosis strategy depended on the surgeon's preference which potentially include either (1) partial clamping of the ascending aorta or (2) use of proximal anastomosis assist devices, including Enclose II (Vitalitec International Inc., Plymouth, MA, USA) and HEARTSTRING III (MAQUET Holding B.V. & Co. KG, Rastatt, Germany).

### 2.4 Statistical analysis

Group assignments were not random as the operative approach was a matter of subjective choice. Therefore, we calculated the standardized mean differences before and after PSM to assess the balance of variables between the groups. The propensity score (PS) was obtained from a logistic regression model, including eight covariables (age, male sex, presence of diabetes mellitus, two-vessel disease, or three-vessel disease, on dialysis, elective case, and urgent case). Patients were matched in a 1:1 manner using the nearest neighbor matching method without replacement and a caliper width of 0.2 of the standard deviation of the logit of the estimated PS. Parametric quantitative data were expressed as mean + standard deviation, whereas non-parametric data were expressed as median and range. For the comparison of continuous variables exhibiting a normal distribution between two groups, the independent student *t*-test was used, and for continuous variables exhibiting a non-normal distribution, the Mann–Whitney *U*-test was used. Categorical variables were expressed as frequency and percentages and were compared using chi-squared or Fisher's exact test. Statistical significance was set at *p* < 0.05. A univariable or multivariable Cox regression analysis was used to identify the independent risk factors of long-term death, and was presented as hazard ratios (HRs) with 95% confidence intervals. Any variable showing a *P* value of <0.1 in the univariable analysis was included in the multivariable analysis. Multicollinearity tests of independent factors were performed before conducting the multivariable analysis. Kaplan–Meier analysis was conducted to study the survival rate and freedom from MACCE. STATA software/MP, version 17.0 (Stata Corporation, College Station, Texas, USA), was used for all statistical analyses.

## 3 Results

In total, 598 elderly patients with multivessel coronary artery disease aged 70 years who underwent surgical coronary revascularization from August 2017–July 2023 were screened for study eligibility. Among these patients, 162 patients who had either SVG only or SVG with more than two arterial grafts, and eight patients with poor preoperative conditions (emergent or salvage cases and those with Society of Thoracic Surgeons score of >20) were excluded. Finally, the unmatched TAR and LIMA + SVG groups comprised 101 and 327 patients, respectively.

Table 1 summarizes the preoperative characteristics of the studied patients. Before PSM, the preoperative status of the LIMA + SVG group was worse than that of the TAR group. The Society of Thoracic Surgeons (*p* = 0.029) and European System for Cardiac Operative Risk Evaluation (*p* = 0.023) scores were significantly higher in the LIMA + SVG group. After matching, all variables had a standardized mean difference of <10%.

**Table 1:** Patient's characteristics and preoperative data.

Patient's characteristics and preoperative data	Entire cohort			PS-matched cohort		
	TAR group (*n* = 101)	LIMA + SVG group (*n* = 327)	SMD	TAR group(*n* = 100)	LIMA + SVG group (*n* = 100)	SMD
Age, Mean ± SD years	74.1 ± 3.5	75.2 ± 4.2	−0.29	74.1 ± 3.5	73.7 ± 3.6	0.10
Male gender, *n* (%)	70 (69.3)	178 (54.4)	0.30	69 (69.0)	68 (68.0)	0.02
STS SCORE, [IQR]	2.1 (1.5–3.5)	2.9 (1.7–5.0)	−0.21	2.1 (1.5–3.5)	2.2 (1.4–4.1)	−0.04
Euro SCORE, [IQR]	2.4 (1.5–4.7)	3.2 (2.0–5.7)	−0.17	2.4 (1.5–4.6)	2.7 (1.7–5.7)	−0.10
Comorbidity, *n* (%)						
Hypertension	99 (98.0)	322 (98.5)	−0.04	98 (98.0)	99 (99.0)	−0.08
Diabetes mellitus	36 (35.6)	135 (41.3)	−0.12	36 (36.0)	36 (36.0)	−0.01
Chronic renal disease (Cr ≧ 1.5)	20 (19.8)	74 (22.6)	−0.08	19 (19.0)	22 (22.0)	−0.07
Dialysis	14 (14.0)	53 (16.2)	−0.06	14 (14.0)	15 (15.0)	−0.02
COPD	15 (15.6)	51 (15.6)	−0.01	15 (15.0)	18 (18.0)	−0.08
PAD	23 (22.8)	68 (20.8)	0.04	22 (22.0)	24 (24.0)	−0.04
STEMI	14 (13.9)	45 (13.8)	0.01	14 (14.0)	17 (17.0)	−0.08
Double vessel disease	25 (24.8)	56 (17.1)	0.18	24 (24.0)	21 (21.0)	0.08
Triple vessel disease	76 (75.3)	285 (82.8)	0.18	76 (76.0)	79 (79.0)	−0.08
Echocardiography						
Ejection fraction, ±SD %	50.9 ± 15.4	52.2 ± 16.0	−0.08	50.8 ± 15.5	51.0 ± 16.7	−0.01
Urgency, *n* (%)						
Elective	75 (74.3)	236 (72.2)	0.06	75 (75.0)	76 (76.0)	−0.03
Urgent	26 (24.8)	90 (27.5)	−0.08	24 (24.0)	24 (24.0)	0.01

STS, society of thoracic surgeons, Euro SCORE: European system for cardiac operative risk evaluation, COPD: chronic obstructive pulmonary disease, PAD: peripheral arterial disease, STEMI: ST-elevation myocardial infarction.

The operative data of the paired group are shown in Table 2. After matching, no significant differences in the operative time and complete revascularization rate were observed between the two groups, but the total graft number (2.7 ± 0.8 in the TAR group vs. 3.0 ± 0.8 in the LIMA + SVG group; *p* = 0.004) and mean number of distal anastomoses (3.0 ± 0.9 in the TAR group vs. 3.5 ± 1.0 in the LIMA + SVG group; *p* = 0.006) were higher in the LIMA + SVG group. The aorta no-touch technique was more frequently implemented in the TAR group than in the LIMA + SVG group (93.0% in the TAR group vs. 5.0% in the LIMA + SVG group, *p* < 0.001). The details of the total arterial graft is summarized in Table 3.

**Table 2:** Operative data.

Operative data	Entire cohort			PS-matched cohort		
	TAR group (*n* = 101)	LIMA + SVG group (*n* = 327)	*p*-value	TAR group (*n* = 100)	LIMA + SVG group (*n* = 100)	*p*-value
Operating time, mean ± SD min	237.4 ± 59.2	222.5 ± 55.5	0.021	237.2 ± 59.4	229.8 ± 58.3	0.38
Total grafts number, mean ± SD	2.6 ± 0.8	2.9 ± 0.8	0.012	2.7 ± 0.8	3.0 ± 0.8	0.004
Number of distal anastomoses, mean ± SD	3.0 ± 0.9	3.3 ± 0.9	0.019	3.0 ± 0.9	3.5 ± 1.0	0.006
Endarterectomy, *n* (%)	3 (3.0)	12 (3.7)	1.00	3 (3.0)	2 (2.0)	0.65
Complete revascularization, *n* (%)	81 (80.2)	255 (78.0)	0.64	80 (80.0)	83 (83.0)	0.59
None touch aorta, *n* (%)	94 (93.1)	42 (12.8)	<0.001	93 (93.0)	5 (5.0)	<0.001
Conversion to CPB, *n* (%)	1 (1.0)	6 (1.8)	1.00	1 (1.0)	1 (1.0)	1.00
Left mini-thoracotomy, *n* (%)	9 (8.9)	46 (13.0)	0.18	9 (9.0)	13 (13.0)	0.37

CPB, cardiopulmonary bypass.

**Table 3:** The detail of total arterial graft design.

Arterial graft, *n* (%)	Entire cohort (*n* = 101)	PS-matched cohort (*n* = 100)
Over three arterial grafts	57 (56.7)	57 (57.0)
Two arterial grafts	44 (43.5)	43 (43.0)
LIMA	100 (99.0)	100 (100)
RIMA	76 (75.2)	75 (75.0)
RA	39 (38.6)	38 (38.0)
RGEA	42 (41.6)	42 (42.0)
Graft configuration, *n* (%)		
LIMA, RIMA, RGEA	28 (28.0)	28 (28.0)
LIMA-Y composite RA	17 (17.0)	17 (17.0)
LIMA-Y composite RA, RIMA-I composite RA	14 (14.0)	14 (14.0)
LIMA, RIMA	13 (13.0)	13 (13.0)
LIMA-Y composite RIMA	7 (7.0)	7 (7.0)
LIMA-Y composite RIMA, RGEA	7 (7.0)	7 (7.0)
LIMA, RGEA	5 (5.0)	5 (5.0)
LIMA-Y composite RA, RIMA	4 (4.0)	4 (4.0)
LIMA, RIMA-I composite RA	3 (3.0)	2 (2.0)
RIMA-Y composite LIMA	1 (1.0)	1 (1.0)
RIMA-Y composite LIMA, RGEA	1 (1.0)	1 (1.0)
LIMA-Y composite RA, RGEA	1 (1.0)	1 (1.0)
RIMA-I composite RA	1 (1.0)	0
The target area of arterial grafts, *n* (%)		
Left anterior descending artery	101 (100.0)	100 (100.0)
Diagonal branch	43 (42.6)	43 (43.0)
Circumflex territory	88 (87.1)	87 (87.0)
Right coronary system	68 (67.3)	68 (68.0)

LIMA, left internal mammary artery; RIMA, right internal mammary artery; RA, radial artery; RGEA, right gastroepiploic artery.

More than half of the patients received more than three arterial grafts (56.7% in the entire cohort and 57.0% in the matched cohort), whereas the remaining patients received two arterial grafts (43.5% and 43.0%). In the TAR group, the LIMA was used in almost all cases (99.0% in the unmatched cohort and 100% in the matched cohort), followed by the RIMA (75.2% and 75.0%, respectively), RGEA (41.6% and 42.0%, respectively), and RA (38.6% and 38.0%, respectively). In addition, all patients in the TAR group had the LAD territory covered, and approximately 70% of patients had the RCA territory covered (67.3% and 68.0%, respectively). The most common configuration for TAR was the combination of LIMA, RIMA, and RGEA, which were used in approximately 28% of the unmatched and matched cohorts.

The short-term outcomes of the paired groups are shown in Table 4. After PSM, there were no significant differences in the duration of intensive care unit or hospital stay, major complications, or 30-day mortality between the two groups. The TAR group had a lower transfusion rate than the LIMA + SVG group (66.0% vs. 82%, *p* = 0.010).

**Table 4:** Postoperative short-outcomes.

Postoperative short-term outcomes	Entire cohort			PS-matched cohort			
	TAR group (*n* = 101)	LIMA + SVG group (*n* = 327)	*p*-value	TAR group (*n* = 100)	LIMA + SVG group (*n* = 100)		*p*-value
Median ICU stay [IQR], days	2.0 (1.0–3.0)	2.0 (1.0–3.0)	0.59	2.0 (1.0–3.0)		1.5 (1.0–2.0)	0.34
Median hospital stay [IQR], days	6.0 (5.0–6.0)	6.0 (5.0–8.0)	0.26	6.0 (5.0–6.0)		5.5 (5.0–7.0)	0.67
Early extubation (≦24 h), *n* (%)	85 (84.2)	274 (83.8)	0.90	84 (84.0)		82 (82.0)	0.71
Perioperative transfusion, *n* (%)	67 (66.3)	271 (82.9)	<0.001	66 (66.0)		82 (82.0)	0.01
Median drain contents, ml [IQR]	400 (300–500)	400 (350–510)	0.59	400 (300–510)		400 (500–500)	0.28
30 days mortality, *n* (%)	3 (3.0)	9 (2.8)	1.00	2 (2.0)		0 (0)	0.50
Early term postoperative complications, *n* (%)							
New stroke	1 (1.0)	9 (2.8)	0.46	1 (1.0)		2 (2.0)	1.00
New dialysis	0 (0)	5 (1.5)	0.60	0 (0)		1 (1.0)	0.50
New onset atrial fibrillation/flutter	22 (21.8)	120 (36.8)	0.005	21 (21.0)		33 (33.0)	0.06
Infection of wound	2 (2.0)	0 (0)	0.06	2 (2.0)		0 (0)	0.50
Reoperation of bleeding	2 (2.0)	7 (2.1)	1.00	2 (2.0)		2 (2.0)	1.00

ICU, intensive care unit.

**Figure 2:**
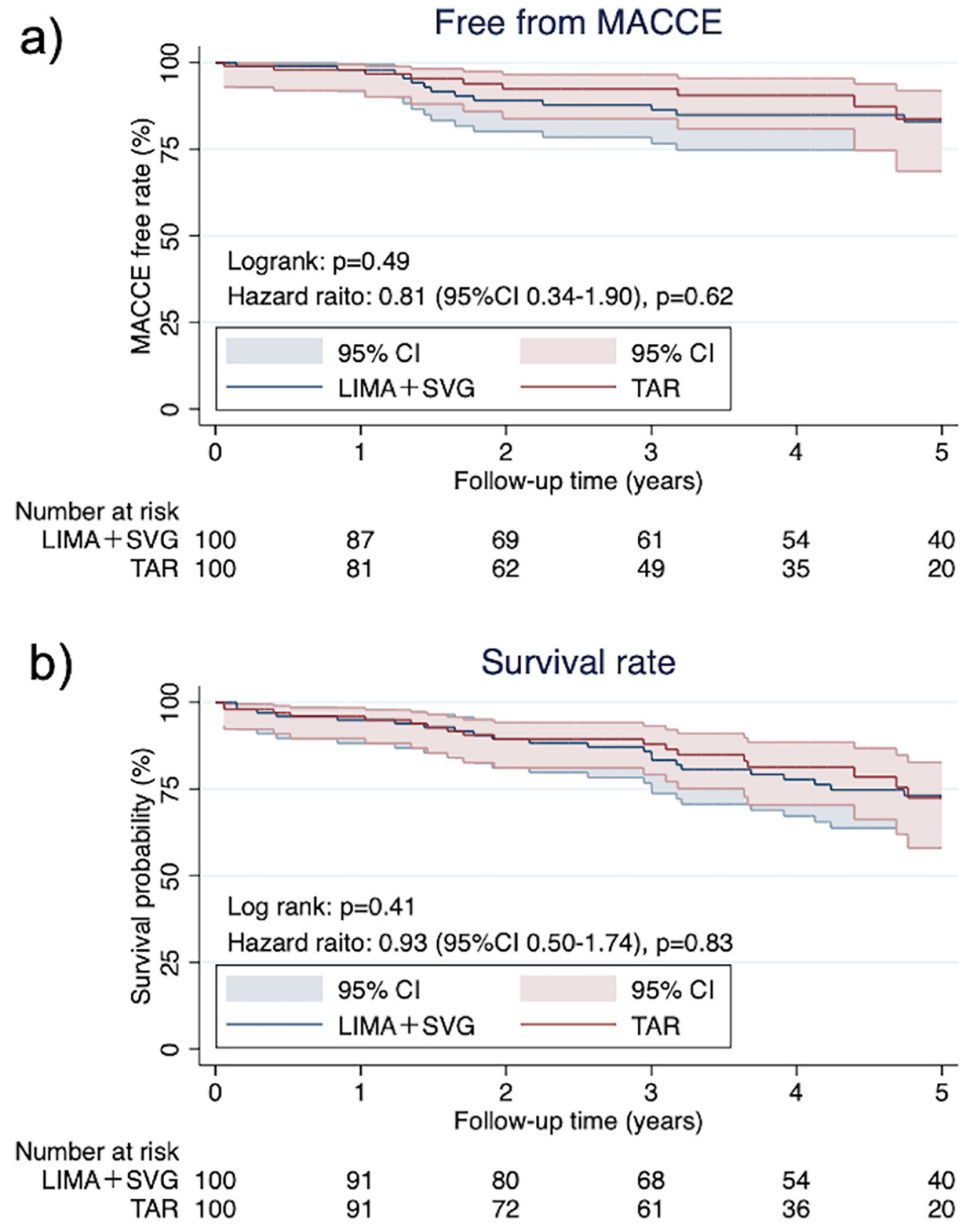
**(a)** MACCE free (*p* = 0.49) and **(b)** survival rate after operations (*p* = 0.41).

After PSM, the median follow-up time was 1,354 [785–2,121] days. The Kaplan–Meier curves of the postoperative free-from MACCE and survival rates are shown in Figure 2. There were no significant differences in both items between the two groups (free-from MACCE rate; *p* = 0.49, survival rate; *p* = 0.41). During the follow-up period, 20 patients died, with the highest occurrence (five cases) observed in those who underwent grafting using the LIMA, RIMA, and GEA.

Table 5 presents the results of the univariable and multivariable regression analyses performed to identify the risk factors for long-term survival. In the univariable analysis, significant associations were detected with diabetes, chronic obstructive pulmonary disease (COPD), high creatinine level (≥1.5 mg/dl), dialysis, peripheral arterial disease (PAD), and preoperative status of either urgent or elective. TAR (HR = 0.74, 95% CI = 0.44–1.22, *p* = 0.23) and complete revascularization (HR = 0.61, 95% CI = 0.34–1.09, *p* = 0.09) showed no significant impact on long-term survival. Multivariate analysis identified diabetes (HR = 1.73, 95% CI = 1.17–2.56, *p* = 0.006), COPD (HR = 1.76, 95% CI = 1.11–2.76, *p* = 0.015), and PAD (HR = 2.32, 95% CI = 1.50–3.60, *p* < 0.001) as significant predictors of long-term survival.

**Table 5:** Univariable and multivariable analyses for factors associated with long-term death.

Variable	Univariable analysis			Multivariable analysis		
	HR	95% CI	*P* value	HR	95% CI	*P* value
Preoperative factor						
Age	1.03	0.99–1.08	0.15	-	-	-
Male gender	1.10	0.75–1.60	0.64	-	-	-
Diabetes mellitus	1.91	1.32–2.78	<0.001	1.73	1.17–2.56	0.006
Chronic renal disease (Cr ≧ 1.5)	2.65	1.79–3.93	<0.001	1.22	0.69–2.16	0.50
Dialysis	2.79	1.85–4.19	<0.001	1.22	0.69–2.16	0.50
COPD	2.43	1.60–3.69	<0.001	1.76	1.11–2.76	0.015
PAD	3.34	2.24–4.99	<0.001	2.32	1.50–3.60	<0.001
STEMI	0.92	0.52–1.60	0.76	-	-	-
DVD	1.02	0.62–1.67	0.95	-	-	-
TVD	0.88	0.55–1.42	0.61	-	-	-
Low EF (<30%)	2.05	1.26–3.32	0.004	1.19	0.71–2.00	0.50
Elective	0.37	0.25–0.54	<0.001	0.38	0.13–1.15	0.09
Urgent	2.52	1.72–3.69	<0.001	0.67	0.22–2.04	0.48
Intraoperative and postoperative factors						
Operation time	1.00	0.99–1.00	0.44	-	-	-
Distal anastomoses number	0.95	0.77–1.17	0.62	-	-	-
TAR	0.74	0.44–1.22	0.23	-	-	-
Complete revascularisation	0.61	0.34–1.09	0.10	-	-	-

## 4 Discussion

Our study included 328 elderly patients, including an unmatched TAR group with 101 patients and unmatched LIMA + SVG group with 327 patients. The PSM groups included 100 patients each. Although off-pump total arterial bypass grafting were performed effectively and safely in the elderly patients with coronary artery disease in our study, it demonstrated no significant effect on the long-term outcomes, as compared with the simple LIMA + SVG procedure.

### 4.1 Active implementation of TAR

Generally, due to the technical difficulties associated with the procedure, the number of grafts and distal anastomoses in TAR is fewer, and the operative time is longer, as compared to conventional OPCAB (9). In our matched cohort, the TAR group had a significantly lower number of distal anastomoses (3.0 ± 0.9 vs. 3.5 ± 1.0, *p* = 0.006) and grafts (2.7 ± 0.8 vs. 3.0 ± 0.8, *p* = 0.004) compared to the LIMA + SVG group. However, complete revascularization was achieved at similar rates (80% vs. 83%, *p* = 0.59). Notably, the operative time did not significantly differ between the two groups (237.2 ± 59.4 vs. 229.8 ± 58.3 min, *p* = 0.38). At our institution, OPCAB is routinely performed, and TAR is selectively employed in patients with favorable conduit anatomy and stable preoperative conditions. Although there were no strict criteria for selecting TAR vs. LIMA + SVG, the decision was made based on intraoperative findings and surgeon judgment. These findings suggest that, when performed by experienced surgeons in high-volume centers, TAR can be safely and effectively applied even in elderly patients without prolonging operative time or compromising revascularization quality.

### 4.2 Impact of TAR on long-term outcomes

The long-term benefits of TAR in CABG have been well-documented in younger patients, and there is strong evidence indicating its association with improved survival and a reduced incidence of MACCE over time (7, 8). However, the evidence in elderly patients remains inconsistent. For example, Zaza et al. analyzed a large registry including both ONCAB and OPCAB patients and found no significant benefit of TAR in those over 70 years or those undergoing OPCAB (10). In contrast, Bisleri et al. demonstrated improved outcomes with OPCAB-TAR in elderly patients during long-term follow-up (11). In our study, TAR in elderly patients undergoing OPCAB did not significantly differ from LIMA + SVG in terms of 5-year survival or free-from MACCE rates. However, since the benefits of TAR often emerge beyond 7–10 years postoperatively (12, 13), the median follow-up duration of approximately 5 years in our study may have been insufficient to capture its long-term advantages. One possible explanation for this finding is the shorter average life expectancy in Thailand than in North America and Europe (14). This demographic difference may have limited the ability to observe the long-term survival benefits of TAR in our cohort. Extended follow-up will be essential to fully assess the impact of TAR in this population.

### 4.3 Study limitations

The present study had several limitations. First, the present investigation is a retrospective, non-randomized analysis using data from a single medical center. Second, we performed PSM based on the different characteristics of the study patients before the operation. Nevertheless, there were several unmeasured confounders. Third, the graft configuration may have influenced the outcomes, which was not fully accounted for in our analysis. Fourth, selection of surgical strategy (TAR vs. LIMA + SVG) was based on surgeon discretion, potentially introducing selection bias. Finally, it is also important to note that the follow-up period in our study was shorter, at only 5 years, as compared to those of previous research. A longer follow-up period is needed to better evaluate the potential long-term benefits of TAR in our specific subgroups.

## 5 Conclusion

Off-pump total arterial bypass grafting for the elderly is effective and safe. However, OPCAB with total arterial grafting did not show a survival and/or free-MACCE advantage in the elderly after 5 years of follow-up, as compared to the procedure using LIMA with SVG.

## Data Availability

The raw data supporting the conclusions of this article will be made available by the authors, without undue reservation.

## References

[1] Lawton JS Tamis-Holland JE Bangalore S Bates ER Beckie TM Bischoff JM 2021 ACC/AHA/SCAI guideline for coronary artery revascularization: a report of the American college of cardiology/American heart association joint committee on clinical practice guidelines. Circulation. (2022) 145(3):e18–e114. 10.1161/CIR.0000000000001038. Erratum in: Circulation. (2022) **145**(11):e772. doi: 10.1161/CIR.0000000000001060. 34882435

[2] Gaudino M Lorusso R Rahouma M Abouarab A Tam DY Spadaccio C Radial artery versus right internal thoracic artery versus saphenous vein as the second conduit for coronary artery bypass surgery: a network meta-analysis of clinical outcomes. J Am Heart Assoc. (2019) 8(2):e010839. 10.1161/JAHA.118.010839 30636525 PMC6497341

[3] Yi G Shine B Rehman SM Altman DG Taggart DP. Effect of bilateral internal mammary artery grafts on long-term survival: a meta-analysis approach. Circulation. (2014) 130(7):539–45. 10.1161/CIRCULATIONAHA.113.004255 24916209

[4] Takagi H Goto SN Watanabe T Mizuno Y Kawai N Umemoto T. A meta-analysis of adjusted hazard ratios from 20 observational studies of bilateral versus single internal thoracic artery coronary artery bypass grafting. J Thorac Cardiovasc Surg. (2014) 148(4):1282–90. 10.1016/j.jtcvs.2014.01.010 24521973

[5] Gaudino M Benedetto U Fremes S Ballman K Biondi-Zoccai G Sedrakyan A Association of radial artery graft vs saphenous vein graft with long-term cardiovascular outcomes among patients undergoing coronary artery bypass grafting: a systematic review and meta-analysis. JAMA. (2020) 324(2):179–87. 10.1001/jama.2020.8228 32662861 PMC7361649

[6] Gaudino M Di Franco A Rahouma M Tam DY Iannaccone M Deb S Unmeasured confounders in observational studies comparing bilateral versus single internal thoracic artery for coronary artery bypass grafting: a meta-analysis. J Am Heart Assoc. (2018) 7(1):e008010. 10.1161/JAHA.117.008010 29306899 PMC5778975

[7] Gaudino M Puskas JD Di Franco A Ohmes LB Iannaccone M Barbero U Three arterial grafts improve late survival: a meta-analysis of propensity-matched studies. Circulation. (2017) 135(11):1036–44. 10.1161/CIRCULATIONAHA.116.025453 28119382

[8] Yanagawa B Verma S Mazine A Tam DY Jüni P Puskas JD Impact of total arterial revascularization on long term survival: a systematic review and meta-analysis of 130,305 patients. Int J Cardiol. (2017) 233:29–36. 10.1016/j.ijcard.2017.02.010 28185702

[9] Vallely MP Seco M Ramponi F Puskas JD. Total-arterial, anaortic, off-pump coronary artery surgery: why, when, and how. JTCVS Tech. (2021) 10:140–8. 10.1016/j.xjtc.2021.09.050 34977717 PMC8691864

[10] Samadashvili Z Sundt TM 3rd Wechsler A Chikwe J Adams DH Smith CR Multiple versus single arterial coronary bypass graft surgery for multivessel disease. J Am Coll Cardiol. (2019) 74(10):1275–85. 10.1016/j.jacc.2019.06.067 31488263

[11] Bisleri G Di Bacco L Turturiello D Mazzoletti A Giroletti L Repossini A Improved outcomes of total arterial myocardial revascularization in elderly patients at long-term follow-up: a propensity-matched analysis. Ann Thorac Surg. (2017) 103(2):517–25. 10.1016/j.athoracsur.2016.06.028 27577035

[12] Taggart DP Gaudino MF Gerry S Gray A Lees B Dimagli A Effect of total arterial grafting in the arterial revascularization trial. J Thorac Cardiovasc Surg. (2022) 163(3):1002–1009.e6. 10.1016/j.jtcvs.2020.03.013 32305186

[13] Ren J Royse C Royse A. Late clinical outcomes of total arterial revascularization or multiple arterial grafting compared to conventional single arterial with saphenous vein grafting for coronary surgery. J Clin Med. (2023) 12(7):2516. 10.3390/jcm12072516 37048600 PMC10094905

[14] Karcharnubarn R Rees P Gould M. Healthy life expectancy changes in Thailand, 2002–2007. Health Place. (2013) 24:1–10. 10.1016/j.healthplace.2013.08.002 23999577

